# Intra-operative Measurement of Eustachian Tube Orifice, and Its Correlation With Post-operative Surgical Outcome in Chronic Suppurative Otitis Media Patients

**DOI:** 10.7759/cureus.68706

**Published:** 2024-09-05

**Authors:** Mohan Raaj, RN Karadi, Lathadevi THT, Manali Ramana Bhat, Shashikumar T

**Affiliations:** 1 Otolaryngology - Head and Neck Surgery, Shri B. M. Patil Medical College Hospital and Research Centre, Bijapur Lingayat District Educational (BLDE) Association (Deemed to be University), Vijayapura, IND

**Keywords:** csom: chronic suppurative otitis media, eustachian tube anatomy, eustachian tube function, graft uptake, tubotympanic type

## Abstract

Background

Chronic suppurative otitis media (CSOM) is the inflammation of the middle ear mucosa for more than two weeks, resulting in ear discharge. It is associated with hearing loss and the presence of a perforation in the tympanic membrane. Tympanoplasty is performed to place a graft and clear the disease in the middle ear. Despite adequate disease clearance and proper graft placement, graft failure and disease persistence occur due to Eustachian tube (ET) dysfunction. The ET plays a significant role in the ventilation of the middle ear. Hence, this study was conducted to determine the significance of ET size for post-operative graft uptake.

Methodology

A total of 55 patients with inactive CSOM were included in the study. Their demographic data were recorded. Patients previously operated on for CSOM, cases with traumatic perforation of the tympanic membrane, congenital anomalies (e.g., cleft lip/cleft palate), and atticoantral disease were excluded. Thorough history taking and examination, including otoscopy and examination of the nose, throat, and oropharynx, were conducted. Once the patient was deemed fit for surgery, they underwent tympanoplasty. Intraoperatively, the ET size was measured using the tip of the suction cannulas. They were followed up after three months to assess graft uptake.

Results

Out of 55 patients included in the study, 42 (76%) had good graft uptake, while 13 (24%) had defects in graft uptake. Graft uptake failed in patients with an ET diameter of <3 mm. Post-operative graft uptake was observed in the majority of patients with a wider ET diameter, ranging between 3 mm and 6 mm, with a statistically significant p-value of 0.00 (0.05), as determined by Pearson’s Chi-square test.

Conclusion

In our study, we found that there is an association between the ET diameter and post-operative graft uptake. Hence, a wider ET may improve middle ear ventilation and play an important role in post-operative graft uptake.

## Introduction

Chronic suppurative otitis media (CSOM) is the inflammation of the middle ear mucosa for more than two weeks, resulting in ear discharge. CSOM has two types: tubotympanic (mucosal/safe type) and atticoantral (squamosal/unsafe type). If left untreated, it may result in hearing loss. Eustachian tube (ET) function has been the centre of focus as a prognostic factor. The ET is a part of the middle ear that connects the nasopharynx to the middle ear. It is approximately 35 mm long. The three major functions of the ET in the middle ear are that it protects sound pressure from the nasopharynx, helps to drain middle ear secretions, and maintains equilibrium between the air pressure in the middle ear and the atmosphere [1]. Closing of the ET protects the middle ear from pressure fluctuations. It was believed that tubal dysfunction is one of the most important factors for the failure of tympanoplasty [2]. Reconstructive ear surgery, especially tympanoplasty, is one of the most common surgeries performed by otologists. The success of the surgery is indicated by improvement in hearing, non-persistence of the symptoms, and post-operative graft uptake. One of the important factors on which the success of the surgery depends is the proper functioning of the ET [3].

Infection from the nasopharynx travels through the ET, resulting in the mucosal type of chronic otitis media. Many studies have emphasized ET function using functional tests, like impedance audiometry, Toynbee's test in patients with a perforated tympanic membrane, and William’s test in patients with a normal tympanic membrane. Based on the pre-operative ET function, patients were selected for tympanoplasty. In a few studies, other methods, such as dynamic slow-motion video endoscopy, were used to assess ET function. Many studies have focused mainly on ET function, but in our study, emphasis has been placed on directly measuring the intra-operative ET orifice diameter and comparing it with the post-operative surgical outcome in the form of graft uptake.

## Materials and methods

Source of data

It is a hospital-based prospective study. The study duration was between November 2022 and June 2024. A total of 55 patients were included in the study. The Ethical Clearance Committee of Shri B. M. Patil Medical College Hospital and Research Centre, BLDE (Deemed to be University), Vijayapura, India, issued approval with IRB Number: BLDE(DU)/IEC/708/2022-2023.

Inclusion criteria

All cases of inactive CSOM (mucosal), attending the ENT OPD from November 2022 to June 2024, and undergoing type-I tympanoplasty at Shri B. M. Patil Medical College Hospital and Research Centre, were included in the study.

Exclusion criteria

Patients previously operated on for CSOM, those with traumatic perforation of the tympanic membrane or atticoantral disease, and patients with cleft lip/cleft palate were excluded from the study.

Method of collection of data

A total of 55 patients were included in the study. Their demographic data were recorded. Thorough history taking and examination, including otoscopy, were done to determine the site and size of the perforation. A detailed examination of the nose, throat, and oropharynx was also conducted. If discharge was present, patients were administered oral or IV antibiotics, along with a nasal decongestant, until the ear was dry. Tips of the Frazier/Lempert nasal suction cannulas, of various diameters ranging from 3 mm to 6 mm, were used to measure the intra-operative ET diameter. These suction cannulas' tips were introduced into the ET orifice to measure its diameter. They were followed up after three months to assess the post-operative graft uptake.

Statistical analysis

The descriptive summary was done for all variables. Data were analyzed using the Chi-square test or Fisher’s exact test for association, comparison of means, sensitivity, specificity, and diagrammatic presentation. The results were considered statistically significant if the p-value was less than 0.05.

## Results

A total of 55 patients with an inactive mucosal type of CSOM were included in the study. Out of these 55 patients, most were between the ages of 30 and 39. Additionally, 21.8% of patients were in the age group of 20-29. There was an almost equal gender distribution among the patients included in the study. Of the 55 patients enrolled, 31 (56.4%) had right ear disease, and 24 (43.6%) had left ear disease. Out of the 55 patients, 48 (87.3%) had complaints of reduced hearing, while seven (12.7%) had no such complaints. This indicates that the majority of patients had reduced hearing associated with CSOM. Furthermore, among the 55 patients, 11 (20.0%) had large central perforation (LCP), 19 (34.5%) had medium central perforation (MCP), 19 (34.5%) had small central perforation (SCP), and six (10.9%) had subtotal perforation. The majority of patients presented with SCP to MCP (Table 1).

**Table 1:** Demographic distribution of data SCP: Small central perforation; MCP: Medium central perforation; LCP: Large central perforation

Age (in years)	No. of patients	Percentage
<20	7	12.7
20-29	12	21.8
30-39	13	23.6
40-49	10	18.2
50-59	9	16.4
60+	4	7.3
Sex		
Female	27	49.1
Male	28	50.9
Ear discharge		
Right	31	56.4
Left	24	43.6
Reduced hearing		
Yes	48	87.3
No	7	12.7
Tympanic membrane (size of perforation)		
LCP	11	20.0
MCP	19	34.5
SCP	19	34.5
Subtotal	6	10.9
Total	55	100.0

**Figure 1:**
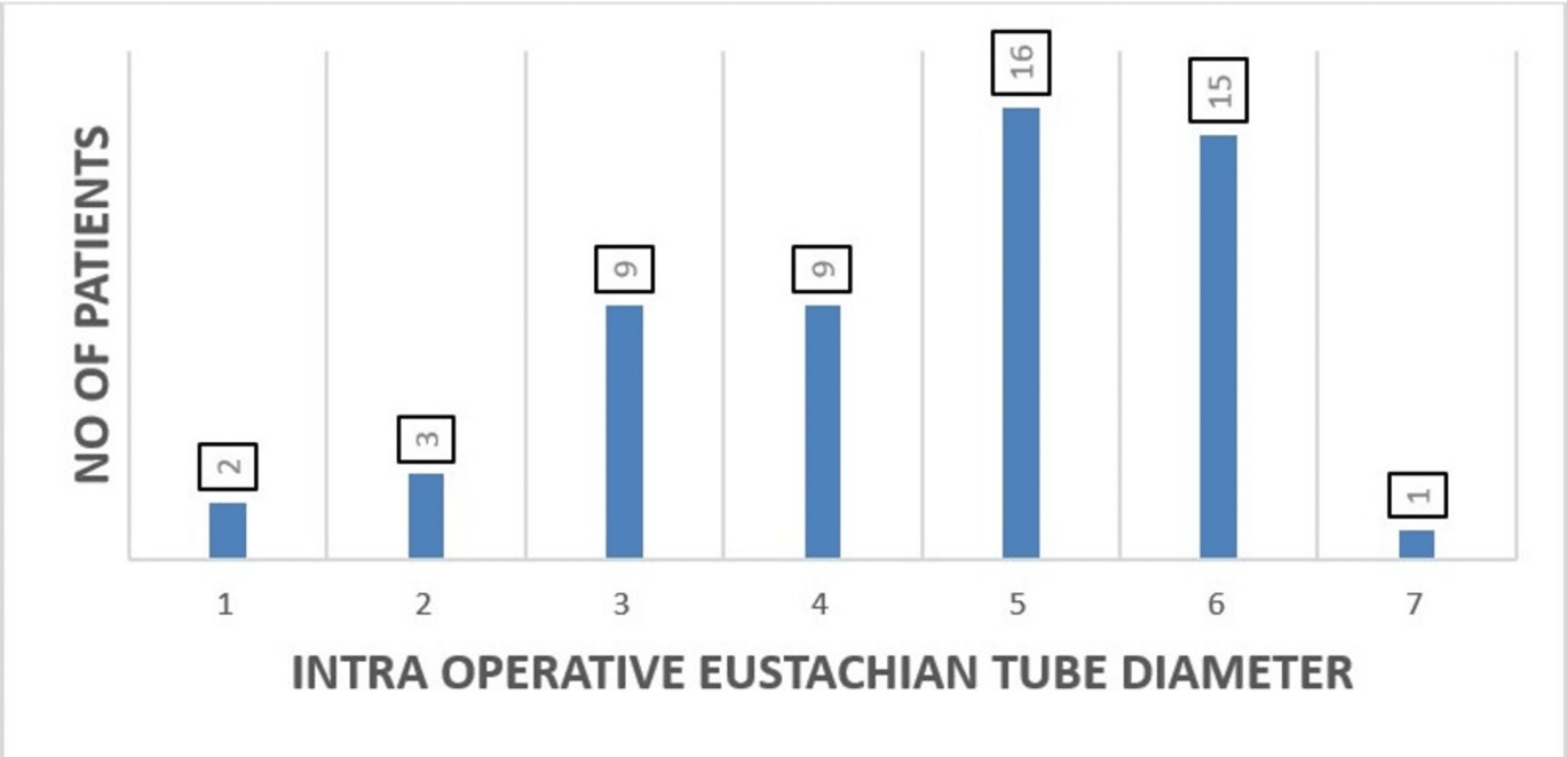
Distribution according to intra-operative Eustachian tube diameter

Out of 55 patients enrolled in the study, 16 (29.1%) had an intra-operative ET diameter of 5 mm, followed by 15 (27.3%) patients with an ET diameter of 6 mm. Nine (16.4%) patients had a tube diameter of 4 mm, and nine (16.4%) patients had a tube diameter of 3 mm. The remaining patients had tube diameters of 2 mm and 1 mm (Table 2; Figure 1).

**Table 2:** Distribution according to intra-operative Eustachian tube diameter

Intra-operative Eustachian tube diameter (in mm)	No. of patients	Percentage (%)
1	2	3.6
2	3	5.5
3	9	16.4
4	9	16.4
5	16	29.1
6	15	27.3
7	1	1.8
Total	55	100

**Figure 2:**
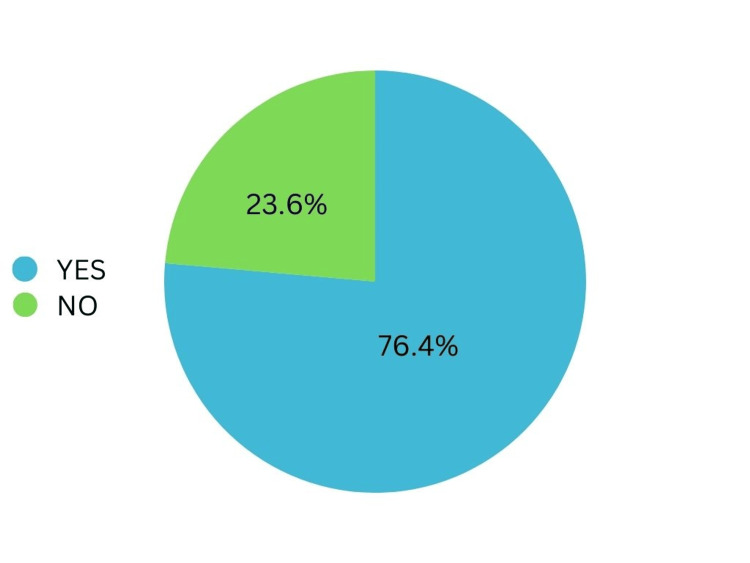
Post-operative graft uptake

Out of 55 patients in the study, 42 (76.4%) had graft uptake, whereas 13 (23.6%) had a graft defect, as shown in Figure 2.

Overall, post-operative graft uptake was seen in 42 (76%) patients. Of these 42 cases with good graft uptake, 26 (61.9%) patients had intra-operative ET diameters between 3 mm and 5 mm, while 16 (38.1%) patients had intra-operative ET diameters greater than 6 mm. Defective graft uptake was observed in 13 (24%) patients. Among these 13 patients, five (38.5%) had intra-operative ET diameters less than 3 mm, and eight (61.5%) had diameters between 3 mm and 5 mm. Thus, patients with wider ET diameters ranging between 3 mm and 6 mm had better graft uptake, with a statistically significant p-value of 0.00 (≤0.05) (Table 3).

**Table 3:** Correlation between intra-operative Eustachian tube diameter and post-operative graft uptake at three months Chi-square value: 21.106; asymptotic significance (p-value): 0.00 (p < 0.05), indicating statistical significance

Intra-operative ET diameter (mm)	Post-operative graft uptake at three months		Total
-	Yes	No	-
<3	0 (0.00%)	5 (38.5%)	5 (9.1%)
3-5	26 (61.9%)	8 (61.5%)	34 (61.8%)
>5	16 (38.1%)	0 (0.00%)	16 (29.1%)
Total	42 (100%)	13 (100%)	55 (100%)

Overall, post-operative graft uptake was seen in 42 (76%) patients. Of these 42 cases with good graft uptake, seven (16.7%) patients had LCP, 16 (38.1%) patients had MCP, 18 (42.9%) patients had SCP, and one (2.4%) patient had subtotal perforation. Defective graft uptake was seen in 13 (24%) patients. Of these 13 patients, four (30.8%) had LCP, three (23.1%) had MCP, one (7.7%) had SCP, and five (38.5%) had subtotal perforation. The significance between the size of perforation and graft uptake was studied using the Chi-square test, which showed a statistically significant p-value of 0.001 (Table 4).

**Table 4:** Correlation between the size of perforation and post-operative graft uptake at three months Chi-square test: p-value of 0.001 (p < 0.05), indicating statistical significance SCP: Small central perforation; MCP: Medium central perforation: LCP: Large central perforation

Tympanic membrane (size of perforation)	Post-operative graft uptake at three months		Total
-	Yes	No	-
SCP	18 (42.9%)	1 (7.7%)	19 (34.5)
MCP	16 (38.1%)	3 (23.1%)	19 (34.5)
LCP	7 (16.7%)	4 (30.8%)	11 (20%)
Subtotal perforation	1 (2.4%)	5 (38.5%)	6 (10.9%)
Total	42 (100%)	13 (100%)	55 (100%)

## Discussion

In our study, out of 55 patients, post-operative graft uptake was correlated with the size of the ET, which was measured intraoperatively. We found that patients with an ET diameter greater than 3 mm had good post-operative graft uptake without any defects in the graft.

We directly measured the ET intraoperatively using standardized suction tips as a form of measurement. With this method, the results were comparable to those of studies using other forms of ET measurement. In a study by Priya et al., where post-operative graft uptake was compared with ET function measured by the Toynbee test, 78% of 100 patients had good ET function, and this was reflected in good graft uptake. Similarly, Tadke et al. used impedance audiometry as an ET function assessment tool and found a strong association between ET function and positive post-operative surgical outcomes [1-3]. Additionally, in a study by Li et al. [4], ETF was significantly improved after type I tympanoplasty for CSOM combined with ET dysfunction.

The ET’s physiological functioning and anatomical patency are required to maintain the normal function of the middle ear. The ET plays a key role in the pathogenesis of various conditions involving the middle ear cleft, such as mucosal cases of CSOM and even cholesteatoma [5]. Additionally, the preoperative middle ear mucosal status (normal or inflamed) also plays a role in healing and prognosis. The major mechanism by which the ET helps prevent middle ear disease is by equalizing any negative pressure build-up in the middle ear cleft [6].

Aspects of the ET other than diameter could have been considered. Few studies suggest no correlation at present [7,8], wherein neither the ET angle nor the ET length affected middle ear functions or surgical outcomes. However, there is some evidence that, even in the absence of obvious craniofacial malformations, individuals with congenital defects causing ET dysfunction are more prone to middle ear disease and CSOM. Such dysfunction could involve abnormal patency or functional obstruction of the tube. Retraction pockets and middle ear effusion are also more common in patients with patulous ETs [9-13].

There are many ways of assessing tubal functions, which include initial middle-ear pressure, active tubal function (muscular opening function), passive function (pressure opening and closing levels), and inflationary and deflationary capacity [14]. ET dysfunction is the primary cause of persistent otitis media, especially in older individuals [15]. This indicates that, despite the mode of measurement - be it quantitative or qualitative - the association is significant nonetheless [16].

In the future, therapeutic measures to address this issue, such as ET balloon dilatation and tuboplasty, are avenues that need further research but hold great promise [17].

Limitation

The study had a small sample size. Only mucosal cases of CSOM were included, so the correlation between the ET orifice diameter and post-operative graft uptake could not be assessed in squamosal diseases, including cholesteatoma. Additionally, the study had no control group, which is also a limitation.

## Conclusions

In our study, we found an association between the ET diameter and post-operative graft uptake. In patients with an ET diameter of <3 mm, there was a defect in post-operative graft uptake. In contrast, there was no post-operative defect in the graft in patients with an ET diameter of >3 mm. This indicates that the ET plays an important role in the outcome of surgery in CSOM patients. Most studies have focused on the functional status of the ET diameter. Further research is advised to assess the ET diameter in a larger sample size to establish its value.

## References

[1] Priya K, Karthikeyan P, Coumare VN, Sambandan AP (2012). Evaluation of Eustachian tube function in chronic suppurative otitis media (tubotympanic type) with reference to its treatment outcome. Indian J Otol.

[2] Tadke KR, Lahane VJ, Wakode PT (2017). Role of impedance audiometry in evaluation of Eustachian tube function and its correlation with tympanoplasty surgery outcome: our experience. IOSR JDMS.

[3] Joshi SS, Jagade M, Agarwal S, Ahire D (2012). Tympanometry, a prognostic indicator of myringoplasty with assessment of Eustachian tube function. Int J Otolaryngol Head Neck Surg.

[4] Li R, Wu N, Zhang J, Hou Z, Yang S (2020). Analysis on the correlation between Eustachian tube function and outcomes of type I tympanoplasty for chronic suppurative otitis media. Acta Otolaryngol.

[5] Dwivedi G, Gupta V, Singh Y, Basu A, Upadhyay K, Bhatia R (2022). Evaluation of Eustachian tube function in cases of chronic otitis media by dynamic slow motion videoendoscopy and impedance audiometry. Indian J Otolaryngol Head Neck Surg.

[6] Takahashi H, Hayashi M, Sato H, Honjo I (1989). Primary deficits in Eustachian tube function in patients with otitis media with effusion. Arch Otolaryngol Head Neck Surg.

[7] Yegin Y, Çelik M, Şimşek BM, Olgun B, Karahasanoğlu A, Kayhan FT (2017). The effect of the angle and length of the Eustachian tube on the success rate of cartilage type 1 tympanoplasty. J Craniofac Surg.

[8] Yu Y, Geffen B, McCrary H (2023). Measurements of the pediatric cartilaginous Eustachian tube: implications for balloon dilation. Laryngoscope.

[9] Takasaki K, Takahashi H, Miyamoto I, Yoshida H, Yamamoto-Fukuda T, Enatsu K, Kumagami H (2007). Measurement of angle and length of the Eustachian tube on computed tomography using the multiplanar reconstruction technique. Laryngoscope.

[10] Smith ME, Bance ML, Tysome JR (2019). Advances in Eustachian tube function testing. World J Otorhinolaryngol Head Neck Surg.

[11] Holmquist J, Olén L (1980). Evaluation of Eustachian tube function. J Laryngol Otol.

[12] Bluestone CD, Cantekin EI (1981). Current clinical methods, indications and interpretation of Eustachian tube function tests. Ann Otol Rhinol Laryngol.

[13] Bluestone CD (1983). Eustachian tube function: physiology, pathophysiology, and role of allergy in pathogenesis of otitis media. J Allergy Clin Immunol.

[14] Bylander-Groth A, Stenström C (1998). Eustachian tube function and otitis media in children. Ear Nose Throat J.

[15] Swarts JD, Bluestone CD (2003). Eustachian tube function in older children and adults with persistent otitis media. Int J Pediatr Otorhinolaryngol.

[16] Yücetürk AV, Unlü HH, Okumuş M, Yildiz T, Filiz U (1997). The evaluation of eustachian tube function in patients with chronic otitis media. Clin Otolaryngol Allied Sci.

[17] van Heerbeek N, Ingels KJ, Rijkers GT, Zielhuis GA (2002). Therapeutic improvement of Eustachian tube function: a review. Clin Otolaryngol Allied Sci.

